# Fetal MRI of the supratentorial brain abnormalities: what we should know about ventriculomegaly?

**DOI:** 10.1007/s11604-023-01462-7

**Published:** 2023-07-26

**Authors:** Shigeko Kuwashima

**Affiliations:** Department of Radiology, Dokkyo Medical University, 880, Kitakobayashi, Mibu, 321-0293 Japan

**Keywords:** Fetus, MRI, Ventriculomegaly, Supratentorial

## Abstract

Fetal MRI is performed to evaluate the brain in cases where an abnormality is detected by ultrasonography (US). Fetal MRI has higher contrast resolution than US. Because the fetal brain is dynamic structure, it is important to know the normal appearance of the brain at different gestational age to be better able to identify abnormalities using MRI. Fast imaging sequences to minimize artifact from fetal motion are required. The main sequences used are ultrafast T2 weighted imaging. Similar to pediatric neuroimaging, images are acquired in the axial, sagittal, and coronal planes. T1 weighted image and Gradient echo-planar T2 weighted images are performed to detect hemorrhage. Ventriculomegaly is the most common central nervous system abnormality identified on US. The causes of ventriculomegaly are very heterogeneous and include developmental, destructive, and obstructive processes, or a combination thereof. MRI improves diagnostic accuracy and can be used to evaluate the etiology of the ventriculomegaly. Moreover, MRI can play an important role in detecting additional findings, which may help to focus on patient counseling and management. This review summarizes and illustrates common pattern of ventriculomegaly due to mainly supratentorial abnormalities.

## Introduction

Fetal ventriculomegaly is one of the most frequently diagnosed abnormalities of the central nervous system (CNS) on Ultrasonography (US). Ventriculomegaly can result from developmental, destructive, and obstructive processes or a combination thereof. Fetal MRI offers several advantages over US, including superior contrast resolution. MRI has been used for diagnosing ventriculomegaly and detecting any other CNS or extra-CNS abnormalities due to its significant impact on the overall outcome.

Because the fetal brain is a dynamic structure, it is important to know the normal appearance of the fetal brain at different gestational ages to be better able to identify abnormalities using fetal MRI. This review summarizes and illustrates the common patterns of ventriculomegaly and associated conditions.

### Fetal MRI technique

MR images are acquired during free maternal breathing. If the pregnant woman cannot tolerate lying on her back, the examination can be performed with the pregnant woman lying on her left side.

Because fetal MR imaging is susceptible to fetal motion, it is performed using ultrafast MRI techniques. T2WI uses ultrafast T2W sequences known as half-Fourier acquired single-shot turbo spin echo or single-shot fast spin echo. Axial, sagittal, and coronal sets of brain images are acquired. A slice thickness of 3 mm (0 mm gap) is used. T1WI is performed using a spoiled gradient echo sequence to detect hemorrhage, fat, and calcification, which can appear hyperintense. T2-star weighted imaging and susceptibility-weighted imaging (SWI) are used to detect hemorrhages.

### Normal development

**Fig. 1:**
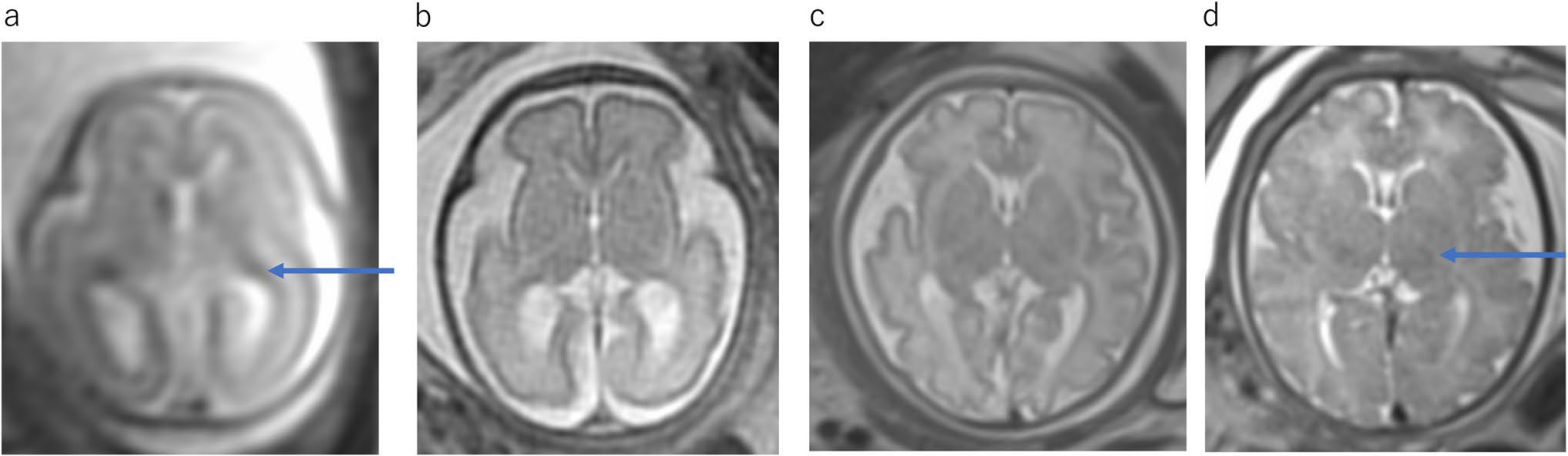
Normal fetal brain development. **a** Normal fetus at 21 gestational weeks. T2WI demonstrates smooth cortical surface and smooth shallow appearance of the sylvian fissures. The germinal matrix appears as a band of low signal surrounding the lateral ventricles (arrow). Lateral ventricles are prominent. **b** Normal fetus at 28 gestational weeks. T2WI demonstrates the configuration of the Sylvian fissures like a box shape. The germinal matrix is less prominent. **c** Normal fetus at 34 gestational weeks. T2WI demonstrates more sulcal formation and reduced size of lateral ventricles. The sylvian fissures have diminished. The cavum septum pellucidum is visible. **d** Normal fetus at 37 gestational weeks. T2WI shows hypointensity in the bilateral ventrolateral thalami corresponding to myelination (arrow)

Before 28 weeks of gestation, the brain is essentially agyric (Fig. 1a, b) [1]. Early in gestation, the ventricles and extra-axial spaces appear prominent relative to the thin cerebral parenchyma. This normal prominence should not be misdiagnosed as ventriculomegaly. A germinal matrix is seen in the walls of the lateral ventricles with low intensity on T2WI (Fig. 1a). Germinal matrix gradually regresses during the third trimester. Sylvian fissures initially appear as smooth, curved, and wide infoldings. After 32 weeks, myelination is observed in the putamen, ventrolateral thalamus, and posterior aspect of the posterior limb of the internal capsule (Fig. 1c, d) [2]. After 37 weeks, the cerebral cortex has thickened, and more sulci have developed [1].

### Ventriculomegaly

The most common indication for fetal brain MRI is ventriculomegaly for CNS abnormalities. Ventriculomegaly can result from developmental, destructive, and obstructive processes or a combination thereof. The atrial diameter of the lateral ventricles is relatively constant during gestation, with normal values being less than 10 mm [2, 3]. Fetal ventriculomegaly can be subclassified into mild (10–12 mm), moderate (12–15 mm), or severe (> 15 mm) [3]. Measurement of the atrial width on MRI images can differ by up to 1 mm to 2 mm compared to US [4]. Neurological outcome is influenced by the severity of ventriculomegaly [3, 4].

The third ventricle can be measured in a coronal image at its largest transverse diameter. A measurement > 4 mm is considered enlarged [2]. The fourth ventricle can be measured on a midline sagittal image from the dorsal pons to the fastigial point. A measurement of more > 7 mm is considered abnormal [2].

**Fig. 2:**
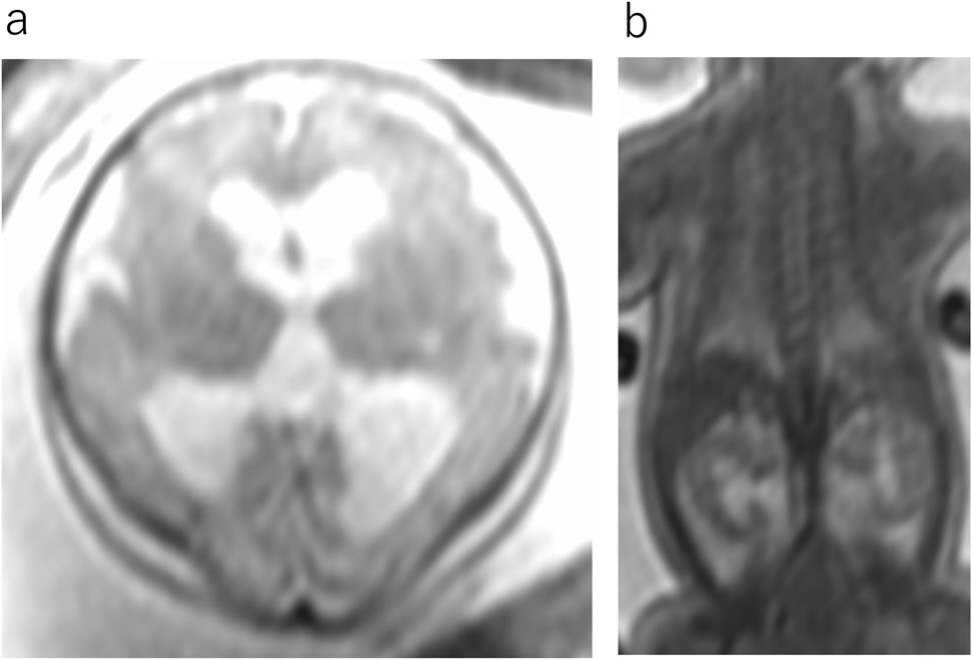
Thanatophoric dysplasia at 32 gestational weeks. **a** Axial T2WI demonstrates bilateral ventriculomegaly. **b** Coronal T2WI shows narrow thorax with short ribs

**Fig. 3:**
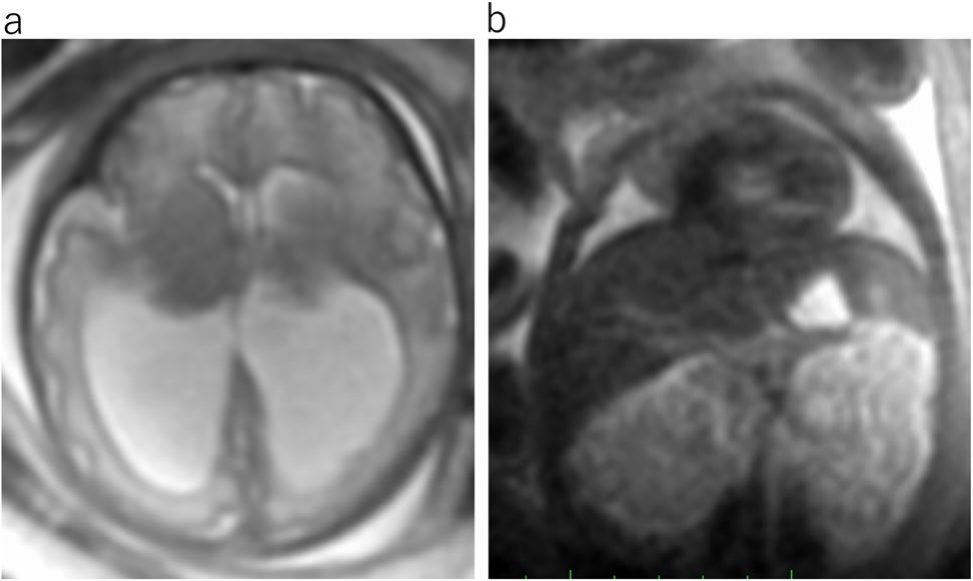
Orofaciodigital syndrome type 1 at 31 gestational weeks. **a** Axial T2WI demonstrates bilateral ventriculomegaly. **b** Coronal T2WI demonstrates that the kidneys are enlarged and loss of parenchymal corticomedullary differentiation

Fetal ventriculomegaly may be the “tip of the iceberg” due to association with other anomalies. An important prognostic factor is whether ventriculomegaly is isolated or associated with additional abnormalities, including chromosomal, CNS and extra-CNS anomalies (Figs. 2, 3).

A systematic approach to fetal MRI evaluation of ventriculomegaly includes evaluation of ventricular morphology, corpus callosum, septum pellucidum, parenchymal signal intensity, sulcation, head size, and extracranial findings.

## Symmetrical ventriculomegaly

### Congenital hydrocephalus

Congenital hydrocephalus can have multiple causes and comprises a diverse group of conditions that result in impaired circulation and absorption of CSF. Increased CSF production is rare and may occur in choroid plexus papillomas.

### Congenital aqueduct stenosis

Congenital aqueduct stenosis (CAS) is a form of obstructive hydrocephalus in which partial or complete obstruction of flow through the aqueduct leads to enlargement of the third and lateral ventricles [5].

**Fig. 4:**
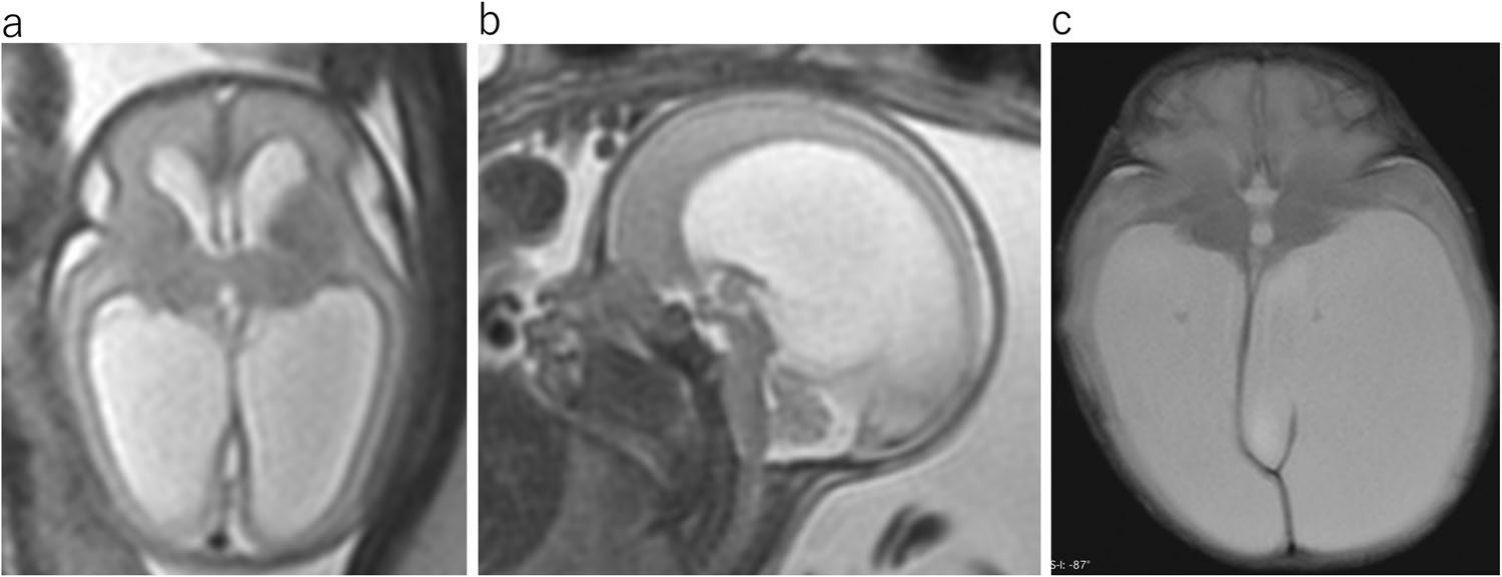
X-linked hydrocephalus due to congenital aqueduct stenosis at 26 gestational weeks (**a**, **b**) and at 35 gestational weeks (**c**). **a** Axial T2WI demonstrates significant ventriculomegaly. **b** Sagittal T2WI shows obstruction of the aqueduct. Lateral ventricle is severe dilatation, and fourth ventricle is normal appearance. **c** Ventriculomegaly is progressive from 26 to 35 gestational weeks

**Fig. 5:**
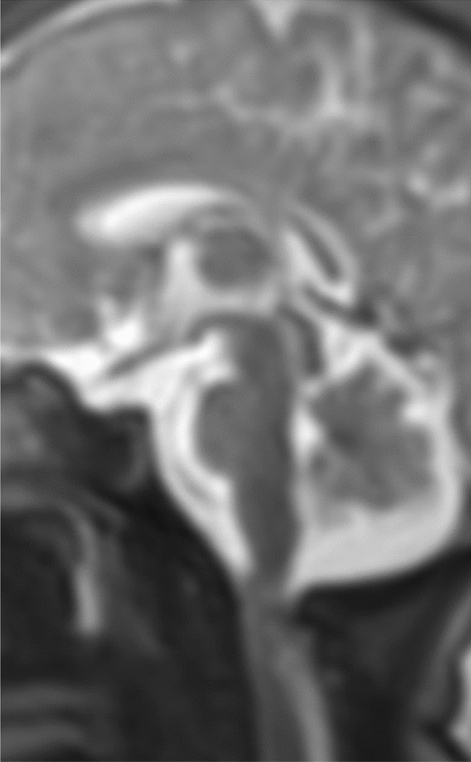
Normal fetus at 36 gestational weeks. Midline sagittal T2WI demonstrates a patent aqueduct and normal morphology of the inferior third ventricle

The MRI findings include enlarged third and lateral ventricles, aqueduct stenosis, and abnormal narrowing of the subarachnoid space (Fig. 4a, b). The size of the fourth ventricle is typically normal. Ventriculomegaly can progress with increasing gestational age (Fig. 4c). On the other hand, Normal aqueduct can be seen on a well—positioned sagittal view of the fetal brain (Fig. 5).

**Fig. 6:**
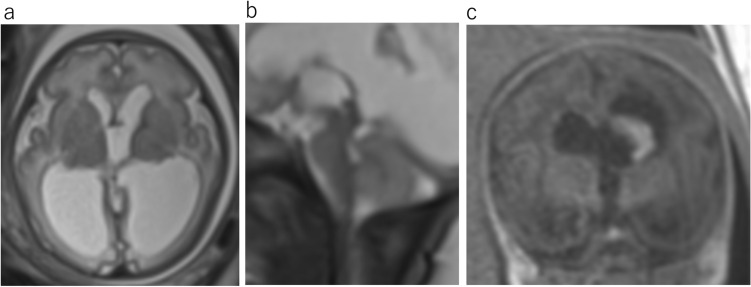
Aqueduct stenosis secondary intraventricular hemorrhage at 34 gestational weeks. **a** Axial T2WI demonstrates lateral and third ventricular enlargement. **b** Sagittal T2WI shows funnel-shaped aqueduct with distal obstruction. Forth ventricle appears normal size. **c** Coronal T1WI shows dilatation of the left lateral ventricle with high intense lesion, consistent with intraventricular hemorrhage

The etiology of CAS is multifactorial, including both genetic and acquired form. Acquired causes commonly result from infection or hemorrhage, with resultant webs or gliosis tissue effacing the cerebral aqueduct (Fig. 6) [3, 5].

There are four described genes, two of which are X-linked (L1CAM and AP1S2) and two are autosomal recessive genes (CCDC88c and MPDZ) [5].

X-linked hydrocephalus is a genetic syndrome caused by a mutation in the L1CAM gene on the X chromosome (Xq28) [3]. L1CAM-associated hydrocephalus is the most common heritable form of ventriculomegaly. It is thought to account for up to 10% of male patients with isolated idiopathic hydrocephalus, particularly in the presence of adducted thumbs [3]. The MRI findings of L1CAM-associated hydrocephalus is characterized by severe dilatation of the third and lateral ventricles, aqueduct stenosis, enlarged quadrigeminal plates, interthalamic adhesion hypertrophy, thinning of the corpus callosum, hypoplasia of the cerebellar vermis, and adducted thumbs (Fig. 4).

Ventriculomegaly in the setting of Rhombencephalosynaosis (RES) or dystroglycanopathy has also been shown to occur because of congenital aqueduct stenosis [5].

**Fig. 7:**
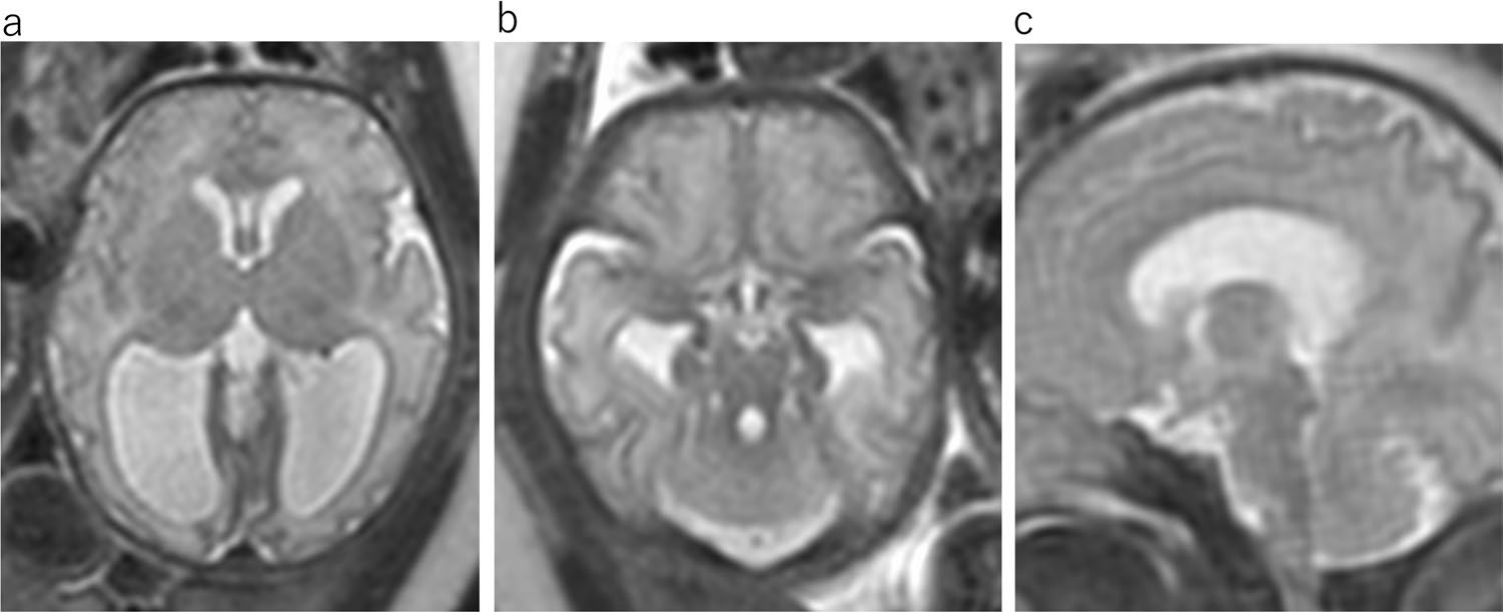
Rhombencephalosynapsis at 33 gestational weeks. **a** Axial T2WI shows dilatation of the bilateral posterior horn of lateral ventricular. **b** Axial T2WI shows continuity of the cerebellar folia without an intervening vermis and “keyhole-shaped” fourth ventricle. c sagittal T2WI shows aqueduct stenosis

**Fig. 8:**
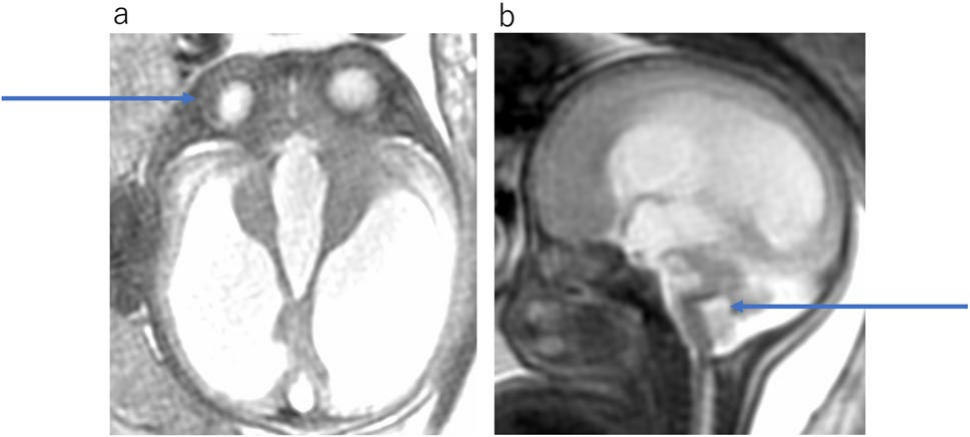
Walker-Warburg syndrome at 35 gestational weeks. **a** Axial T2WI shows severe bilateral ventriculomegaly and right microphthalmia (arrow). **b** Sagittal T2WI shows pontine hypoplasia and a kinked or Z-shaped brainstem (arrow)

RES is a rare cerebellar malformation characterized by vermian agenesis or hypogenesis and apparent fusion of cerebellar hemispheres. The fusion of the cerebellar hemisphere is not caused by maldevelopment of the vermis but probably by primary failure of vermian differentiation [6]. Aqueduct stenosis, which may cause congenital hydrocephalus, is commonly associated with this anomaly. MR findings include fusion of the cerebellar hemisphere with transversely oriented folia and continuity of the cerebellar white matter without formation of the cerebellar vallecula, hypogenesis or agenesis of the cerebellar vermis, and keyhole-shaped or diamond-shaped fourth ventricle (Fig. 7) [7]. The clinical spectra of RES depend mostly on supratentorial anomalies [8]. Typical clinical manifestations include: truncal ataxia, limb ataxia, muscular hypotonia, spasticity, and abnormal eye movement. The severity of neurological dysfunction ranges from mild to severe.α- Dystroglycanopathies are a heterogeneous group of autosomal recessive disorders associated with muscular dystrophies resulting from mutations in some genes responsible for the glycosylation of α-dystroglycan. The muscles, brain, and eyes are usually affected byα-dystroglycanopathies [7]. These diseases vary in severity from mild adult-onset muscular dystrophy to more severe phenotypes with early-onset and brain involvement. Walker-Warburg syndrome is the most severe type of malformation[7]. Walker-Warburg syndrome presents at birth with generalized hypotonia, muscle weakness, developmental delay, mental retardation, and ocular abnormalities. MRI findings include a diffusely abnormal cerebral cortex, hydrocephalus, variable callosal hypoplasia, a very small cerebellar hemisphere, and vermis (Fig. 8). The pons is hypoplastic, and the brainstem is kinked posteriorly at the pontomesencephalic junction (Fig. 8). Brainstem kinking is often detected by fetal MRI and is a characteristic finding ofα-dystroglycanopathies. However, this finding is not pathognomonic. Brainstem kinking has been reported in α-dystroglycanopathy, X-linked hydrocephalus, and tubulinopathy [7].

### Agenesis of the corpus callosum (ACC)

**Fig. 9:**
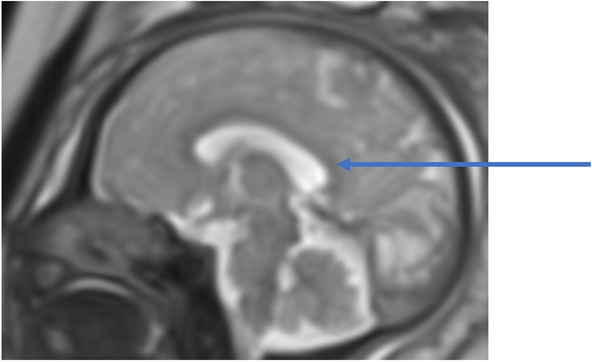
Normal fetus at 30 gestational weeks. Sagittal T2WI shows normal appearance of corpus callosum, showing hypo intensity (arrow)

The corpus callosum is a midline cerebral structure located at the superior margin of the cavum septum pellucidum. It is the largest cerebral commissural connection in the cerebral hemisphere. Beginning at eight weeks of gestation, it assumes its final shape by 20 weeks [1]. The corpus callosum increases in length with increasing gestational age. It is seen as a curvilinear T2 hypointense structure in the midsagittal section from 18 weeks of gestation (Fig. 9) [4]. If the normal developmental process is disturbed, it may be completely absent (agenesis) or partially formed (hypogenesis) [9]. However, even if the callosal precursor forms normally, some insults that disturbs the growth of the fibers may cause callosal hypoplasia.

**Fig. 10:**
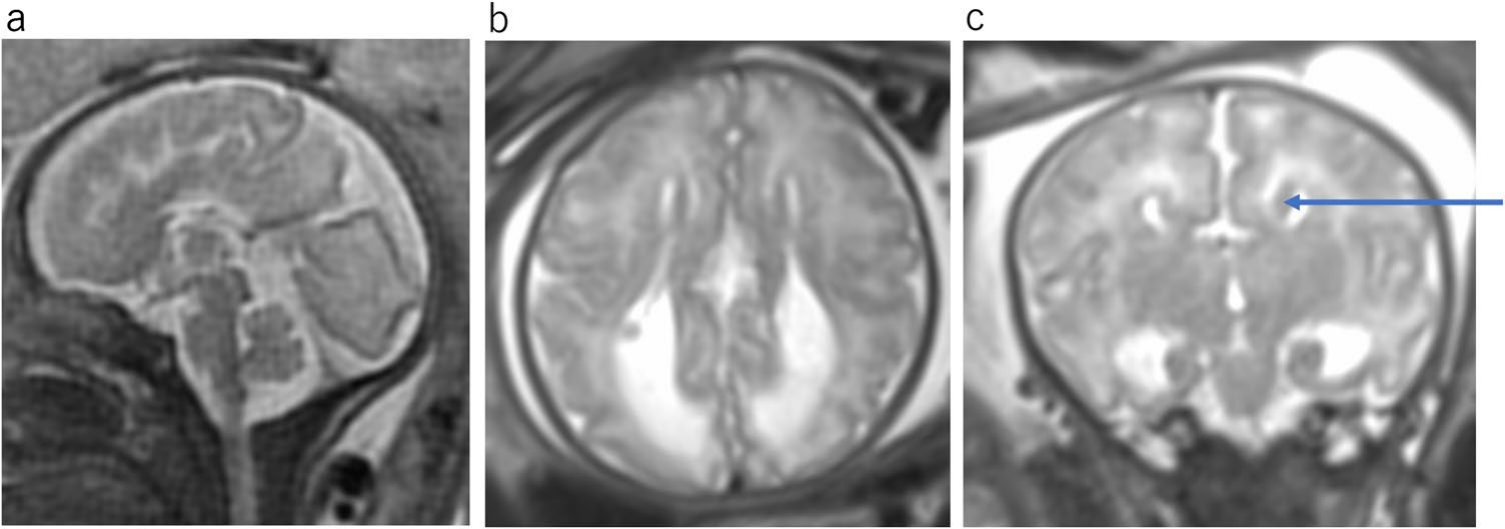
Agenesis of corpus callosum at 33 gestational weeks. **a** Sagittal T2WI shows absent corpus callosum and radiating parasagittal gyri. **b** Axial T2WI shows the parallel bodies of the lateral ventricles and an enlarged posterior horn of the lateral ventricles. **c** Coronal T2WI reveals widely spaced lateral ventricles and the “steer-horn” configuration of the anterior horns with Probst bundles (arrow). The cavum septum is absent

ACC is one of the most common congenital abnormalities of the brain. MR findings are characteristic, with the absence of the corpus callosum and cingulate gyrus with radiating parasagittal gyri (Fig. 10a). Associated findings include colpocephaly, under-rotation of the hippocampi, a parallel configuration of the lateral ventriculus on the axial image (Fig. 10b), a steer-horn configuration of the frontal horn, high-riding third ventricle, and absence of the cavum septum pellucidum on the coronal image (Fig. 10c). Unable to cross the midline, the corpus callosum fibers reroute parasagittal in a bundle commonly known as Probst bundles. The bundles of Probst in the medial walls of the lateral ventricles demonstrate low intensity on T2WI (Fig. 10c).

The cavum septum pellucidum is an important midline forebrain landmark, and its absence often includes additional malformations such as callosal dysgenesis, septo-optic dysplasia, and holoprosencephaly [10].

**Fig. 11:**
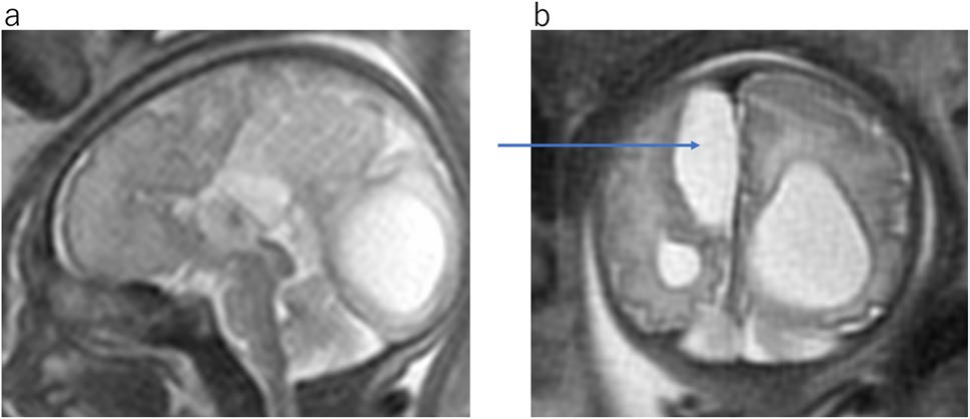
Agenesis of corpus callosum and interhemispheric cyst at 32 gestational weeks. **a** Sagittal T2WI shows absent of the corpus callosum. **b** Coronal T2WI reveals cortical dysplasia around the right interhemispheric cyst(arrow)

ACC can be associated with an interhemispheric arachnoid cyst and/or cystic meningeal dysplasia (Fig. 11) [9]. A diagnosis of isolated ACC implies a relatively benign prognosis. However, some forms of syndromic ACC or its associated anomalies have poor developmental outcomes. There are many types of syndromes and genetic conditions associated with ACC.

**Fig. 12:**
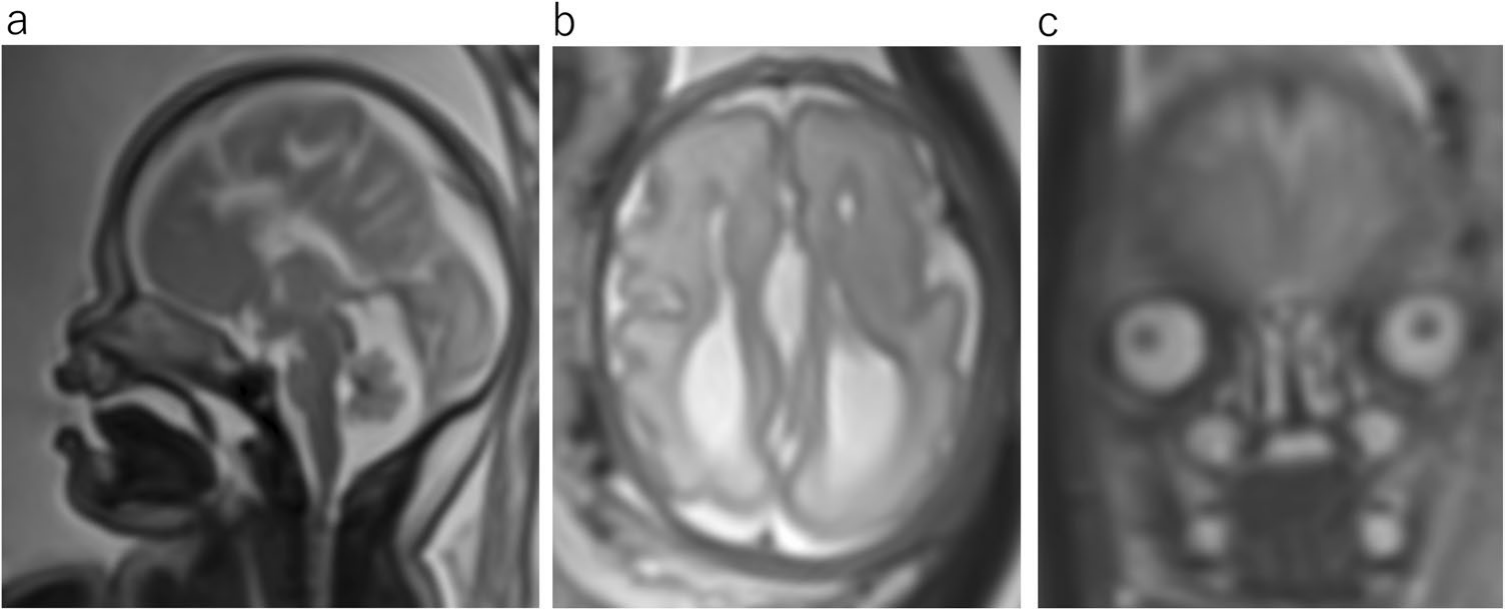
Frontonasal dysplasia sequence at 29 gestational weeks. **a** Sagittal T2WI shows absent corpus callosum. **b** Axial T2WI shows colpocephaly and interhemispheric cyst. **c** Coronal T2WI of the face shows hypertelorism

Craniofrontonasal dysplasia is a rare X-linked developmental malformation caused by a mutation in the *EFNB1*. The most common phenotypic features are hypertelorism, coronal craniosynostosis, frontal bossing, and agenesis of the corpus callosum (Fig. 12) [11].

### Hydranencephaly

**Fig. 13:**
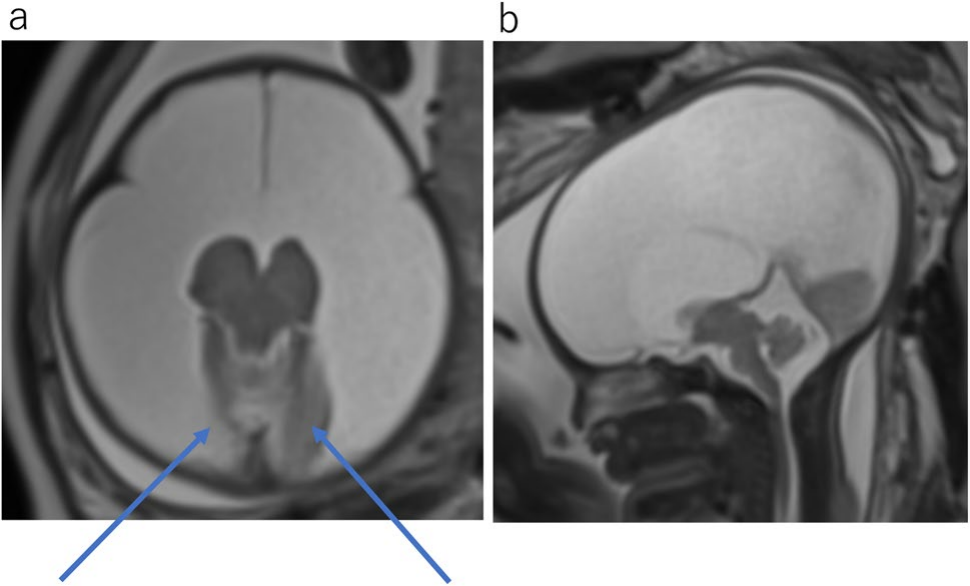
Hydranencephaly at 31 gestational weeks. **a** Axial T2WI shows that the supratentorial compartments are almost filled with CSF and only a small portion of bilateral medial occipital lobes remain (arrows). A falx is present. **b** Sagittal T2WI shows almost all the supratentorial spaces fills CSF with macrocephaly

Hydranencephaly occurs when liquefaction necrosis occurs primarily in the cerebral cortex supplied by anterior circulation. The cerebral hemispheres are largely replaced by thin-walled sacs containing CSF and a complete or near absence of the cerebral cortex (Fig. 13). Because hydranencephaly occurs after the formation of brain structures, the falx remains intact, the thalami are separated, and macrocephaly is because of the persistent production of CSF [12]. The cerebellum is typically intact. The etiology of hydranencephaly is multifactorial, including intrauterine infection, toxic exposure, and events resulting in hemorrhage. Hydranencephaly has rarely described in conjunction with genetic or chromosomal syndromes. Early diagnosis and surgery to avoid problems associated with progressive macrocephaly may improve patient care. The most important differential diagnosis is severe hydrocephalus. A thin cortex can be observed in severe hydrocephalus.

### Congenital infection

Central nervous system infections acquired in utero are a significant cause of morbidity and mortality. The TORCH acronym refers to a group of common perinatal infections. Tissue damage caused by infections is related to pathogen-specific endotoxins, host inflammatory responses, and the timing of infection. The developing brain is particularly sensitive to neurotrophic factors.

**Fig. 14:**
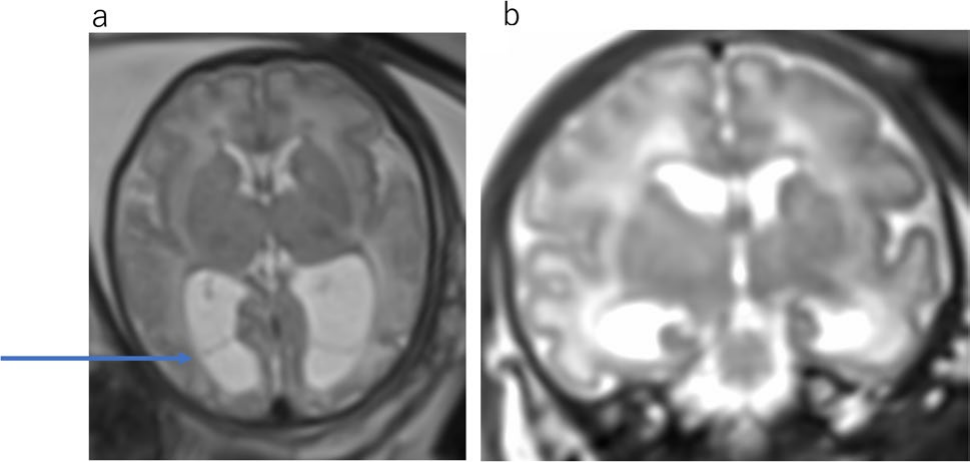
Congenital cytomegalovirus infection at 30 gestational weeks. **a** Axial T2WI shows bilateral ventriculomegaly and occipital intraventricular septum (arrows). **b** Coronal T2WI demonstrates hyperintensities in the central and subcortical white matter

Cytomegalovirus (CMV) is the most common congenital infection. The clinical spectrum of CMV infection is vast, ranging from asymptomatic to severe diseases with multiorgan involvement and mental retardation. Brain imaging findings include cerebral calcification, ventriculomegaly, microcephaly, white matter abnormalities, cortical malformations, cerebellar hypoplasia, subependymal cysts, intraventricular septa, and pathognomonic cystic changes in the occipital or anterior to temporal horns (Fig. 14) [13]. Cysts can occur in and around the ventricular system secondary to necrosis or hemorrhage of the germinal matrix. Microcephaly and cortical anomalies may substantially influence prognosis [13].

## Asymmetrical ventriculomegaly

### Congenital cephalocele

**Fig. 15:**
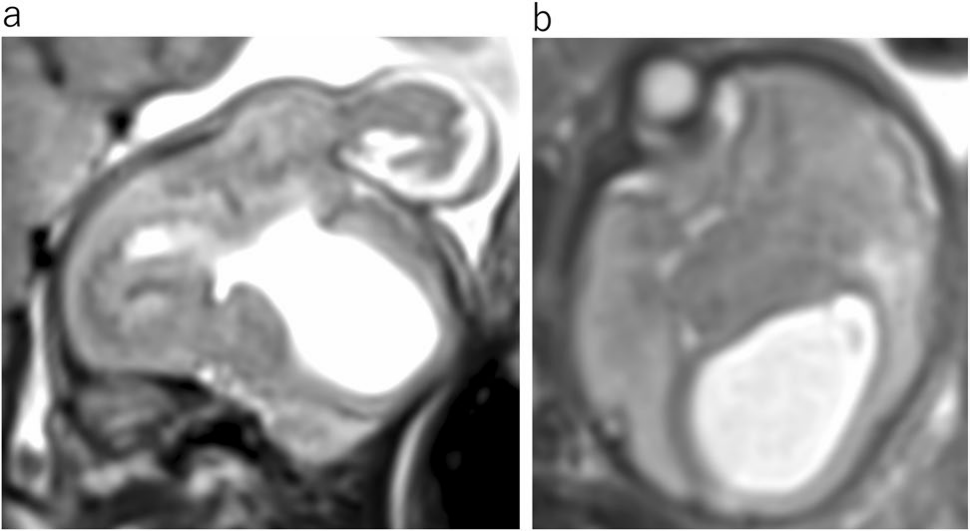
Congenital cephalocele at 27 gestational weeks. **a** Sagittal T2WI shows the huge cephalocele with the presence of neural tissue and CSF in the parietal defect. **b** Axial T2WI shows left ventriculomegaly without normal gyral pattern

Cephalocele occur when there is a skull and dura defect with an extracranial extension of intracranial structures. The prognosis is related to the amount and herniated intracranial contents [5]. The occipital location is the most frequent, followed by the frontal and parietal areas [14]. MRI is useful for evaluating the location of the skull defect and contents of the sac, including herniated dural venous sinuses, and searching for other CNS anomalies (Fig. 15).

### Hemimegalencephaly

**Fig. 16:**
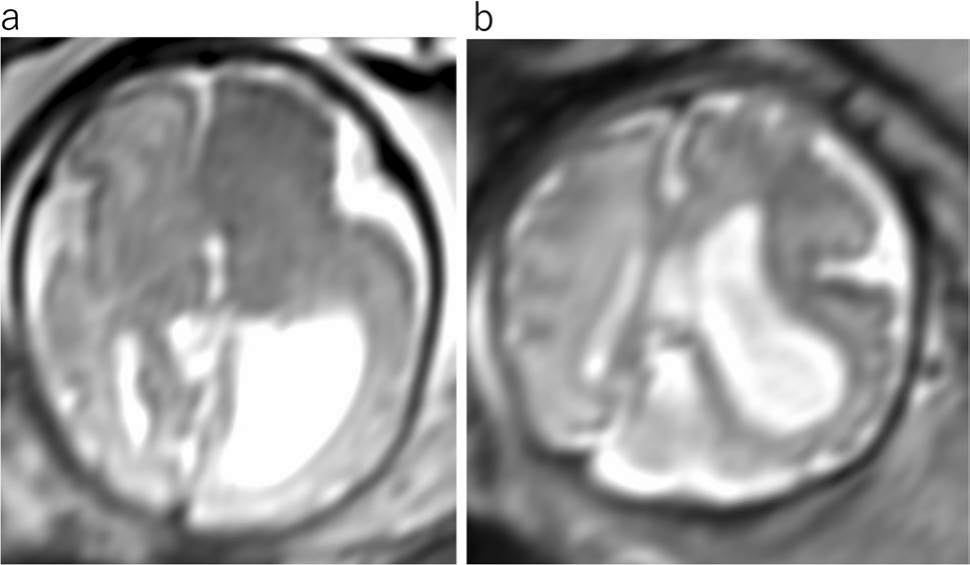
Hemimegalencephaly at 31 gestational weeks. **a** Axial T2WI shows an enlarged left hemisphere and an asymmetric left lateral ventriculomegaly with abnormal gyral pattern, and cortical thickening. **b** Coronal T2WI shows left ventricular enlargement with an abnormal gyral pattern

Hemimegalencephaly is a rare congenital hamartomatous malformation characterized by diffuse or focal enlargement of the cerebral hemisphere. Hemimegalencephaly is a dynamic process, changing from normal size to overgrowth, and can even become atrophic over time. MRI findings of hemimegalencephaly is characterized by an enlarged hemisphere, abnormal gyral pattern, cortical thickening, white matter signal abnormalities, and ipsilateral dilatation of the lateral ventricle (Fig. 16) [9]. The diagnosis of focal hemimegalencephaly can be difficult based on fetal US. Therefore, a fetal MRI is recommended if there is evidence of unilateral ventriculomegaly on US. It is thought that hyperactivation of the mechanistic target of rapamycin (mTOR) pathway is a hallmark of malformation of cortical development such as focal cortical dysplasia or hemimegalencephaly [15]. Extracranial hemi hypertrophy of part or all of the ipsilateral body may be present.

### Post hemorrhagic hydrocephalus

**Fig. 17:**
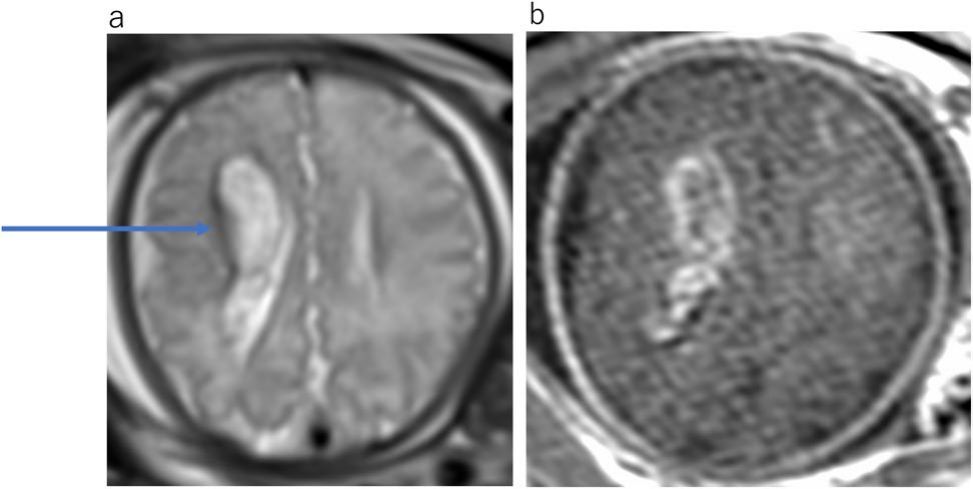
Right subependymal hemorrhage at 34 gestational weeks. **a** Axial T2WI demonstrates abnormal hypointensity along the right lateral ventricle (arrows) with asymmetric right lateral ventriculomegaly. **b** T1WI shows high intensity along the right lateral ventricle extending into the lateral ventricle

A subependymal germinal matrix with intraventricular hemorrhage is a common complication associated with delivery in preterm neonates but rarely occurs in the fetus [3]. The germinal matrix is extremely fragile during the preterm period and is highly susceptible to hypoxemia, anoxia, and changes in blood pressure associated with delivery. However, the pathophysiological conditions of fetal subependymal germinal matrix hemorrhage remains unclear. Mutation in type IV collagen is an important cause of intracerebral hemorrhage. COL4A1 and COL4A2 genes code for the most abundant type IV collagen. Mutations of these genes have been associated with fetal cerebral small vessel disease with intraventricular hemorrhage [16]. In some cases, US can reveal ventriculomegaly but not intraventricular hemorrhage, probably because of its small size or artifacts from the bone. MRI demonstrate ventriculomegaly with parenchymal hemorrhage adjacent to the subependymal and intraventricular hemorrhage with hypointensity on T2WI and hyperintensity on T1WI (Fig. 17). T2-star or SWI are acquired to detect hemorrhage. However, these sequences are more susceptible to fetal motion.

## Conclusion

Fetal MRI can be a valuable tool to complement US. MRI improves diagnostic accuracy and can be used to evaluate the etiology of the ventriculomegaly. MRI can play an important role in detecting additional findings, which may help to focus on patient counseling and management.
